# Motion-corrected Noise2Noise denoising for structural analysis of cells and tissues with (cryo-)FIB/SEM

**DOI:** 10.1093/bioinformatics/btag765

**Published:** 2026-10-16

**Authors:** Vicente González-Ruiz, José-Jesús Fernández

**Affiliations:** Informatics Department, CeinsaUAL, CINN-CSIC Associated Unit, Almería University, 04120 Almería, Spain; Spanish National Research Council (CINN-CSIC), Asturias Health Research Institute (ISPA), 33011 Oviedo, Spain

## Abstract

**Motivation:**

(Cryo-)FIB/SEM enables 3D ultrastructural analysis of cells and tissues at nanoscale resolution. However, its characteristically low signal-to-noise ratio severely hinders interpretation of the volume data. Noise2Noise (N2N) has emerged as a state-of-the-art denoising paradigm with outstanding performance in 3D electron microscopy—particularly cryo-ET—but its direct application to (cryo-)FIB/SEM remains unfeasible due to the inherent acquisition workflows.

**Results:**

A method to enable N2N denoising in (cryo-)FIB/SEM is introduced. The input volume is split into even and odd slices, followed by the application of motion correction methods to estimate and compensate for the structural differences between the two subvolumes. The resulting motion-corrected subvolumes are then used to train the N2N model, which is ultimately applied to denoise the original volume. Representative (cryo-)FIB/SEM volumes have been used to validate the method and to demonstrate the critical importance of the motion correction stage for volumes with a slice thickness >10 nm.

**Availability and implementation:**

Code is available at https://github.com/microscopy-processing/N2N-REO and https://doi.org/10.5281/zenodo.22303478.

## 1 Introduction

FIB/SEM (Focused Ion Beam—Scanning Electron Microscopy) is a volume imaging technique with outstanding potential in cell biology owing to its unique ability to derive 3D structural information from cells and tissues with unlimited thickness at a few-nanometer intervals (Collinson *et al.* 2023). Under cryogenic conditions, referred to as cryo-FIB/SEM, the structural preservation of the sample in close-to-native conditions is ensured (Schertel *et al.* 2013, Dumoux *et al.* 2023). The technique entails cycles of (i) FIB-milling of a thin layer (5–50 nm, so-called slice thickness) of the sample, followed by (ii) SEM-imaging of the exposed surface, so as to collect stacks of images from several μm-thick volumes of the sample. The acquired data thus contain exquisite details of the cellular architecture at nanoscale resolution. The low signal-to-noise ratio (SNR) inherent to this imaging technique, especially in cryo-FIB/SEM, poses significant challenges for visualization and interpretation, thereby making denoising an essential stage.

The standard denoising method in (cryo-)FIB/SEM is Gaussian filtering (Uwizeye *et al.* 2021, Hintze *et al.* 2024, Ch *et al.* 2025, Velazco *et al.* 2025) despite the overall blur it introduces. The application of more sophisticated methods that better preserve the structural features (e.g. non-local means, anisotropic nonlinear diffusion, and deep-learning-based) still remains scarce (Spehner *et al.* 2020, Kapteijn *et al.* 2022) because of their difficulty of use or computational complexity.

Noise2Noise (N2N) is a self-supervised denoising framework based on training a U-Net neural network using two independent noisy versions of a signal as both the input and the target; the model ultimately learns the expectation value, which represents the underlying noise-free signal (Lehtinen *et al.* 2018). N2N has become the state of the art in other 3D nano-imaging modalities, specifically cryo-electron tomography (cryo-ET), thanks to its striking performance (remarkable noise reduction, exceptional background flattening, and excellent preservation of structural features) under extremely low contrast and low SNR conditions (Buchholz *et al.*  2019a, b). However, (cryo-)FIB/SEM is not directly amenable to N2N because its destructive serial imaging workflows prevent the fulfillment of this central requirement (i.e. independent noisy observations of the same volume). To circumvent this situation, subsampling strategies of a single signal have been proposed to synthesize training pairs for N2N (Huang *et al.* 2021, Lequyer *et al.* 2022).

In this work, we introduce an N2N-based approach to single-volume denoising in (cryo-)FIB/SEM. Since the serially acquired cryo-FIB/SEM images contain independent noise, they are ideal for N2N. The proposed method thus splits the image stack into even and odd images to create the two required noisy versions of the volume. A key problem to address is that the structures within these two split subvolumes may differ non-negligibly, which is detrimental to N2N performance. This inter-volume difference is caused by the relatively large slice thickness in (cryo-)FIB/SEM (typically 10–50 nm). To overcome this issue, we apply motion correction methods to warp and align the structures, making the two subvolumes better-suited for effective N2N application.

## 2 Materials and methods

A self-supervised N2N approach to denoising single (cryo-)FIB/SEM volumes can utilize a subsampling procedure to produce training pairs (Huang *et al.* 2021, Lequyer *et al.* 2022). Therefore, given a noisy volume X, two subvolumes $X^{\left(\text{even}\right)}$ and $X^{\left(\text{odd}\right)}$ are generated from the even and odd slices, respectively. Because the images are acquired independently in the serial imaging workflow of (cryo-)FIB/SEM, these subvolumes provide two independent noisy observations of approximately the same clean signal. The U-Net model is trained with this pair, and the trained model is subsequently used during the inference process to denoise X and thus yield a denoised volume Y. We refer to this approach as N2N-EO.

Nevertheless, the previous procedure has several drawbacks compared to the ideal scenario involving two true noisy instances of the exact same clean signal, as occurs in cryo-ET (Buchholz *et al.*  2019a, b). First, as a consequence of the subsampling (which effectively doubles the slice thickness), there is a potential risk of reduced axial resolution and structural blurring during the inference. Second, because the clean underlying signal is not identical in both subvolumes, the model may learn from inaccurate spatial correspondences and also capture the aliasing effect produced by the subsampling, which in the end, is another source of blurring. In summary, the doubled slice thickness and the structural discrepancies between the two subvolumes may lead to blurred solutions with the N2N-EO approach, particularly at larger slice thickness values.

### 2.1 Motion-corrected N2N approach

To mitigate the spatial blurriness problem outlined above, we propose to estimate and compensate for the structural difference between the two subvolumes (even and odd) generated from the input noisy volume. To this end, we employ motion correction methods to apply slice-wise, non-rigid registration of the subvolumes. Figure 1 sketches our proposed approach, termed N2N-REO (registered even/odd), for denoising (cryo-)FIB/SEM volumes, which is described in detail below.

**Figure 1:**
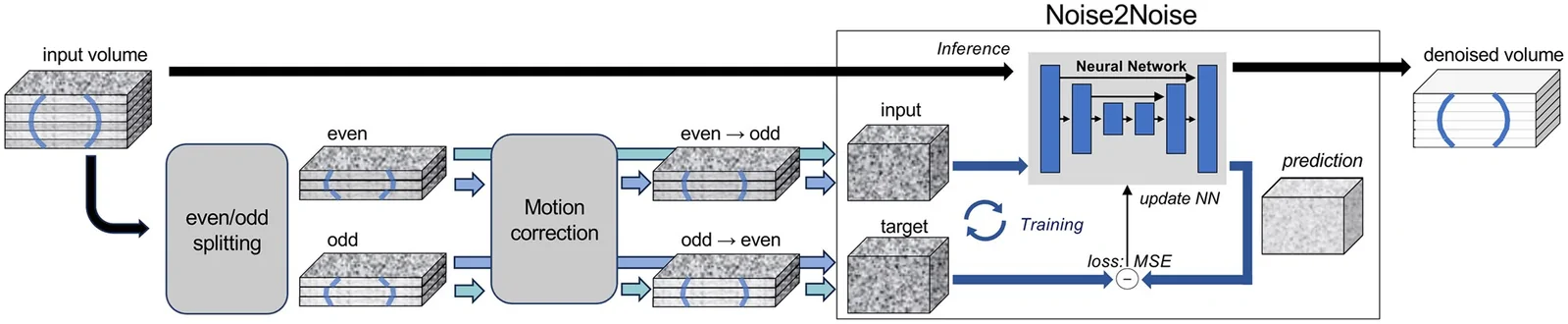
Motion-corrected Noise2Noise denoising for (cryo-)FIB/SEM. The volume is split into two subvolumes (even/odd), which are warped with motion correction to mitigate their differences due to the large slice thickness (even→odd/odd→even). Combinations of the subvolumes are then used to form training pairs ((even, odd→even) and (even→odd, odd), in turquoise and cyan arrows, respectively) to train a U-Net neural network. For the training, the subvolumes are actually split into 3D patches that are randomly selected to serve as the input-target pairs, and the network is updated according to a mean-squared-error-based loss function. Once trained, the network is applied to the original input volume (inference) to yield the denoised version.

To estimate the motion, we utilize dense optical flow (OF) estimation (Farnebäck 2003, González-Ruiz *et al.* 2023). The OF characterizes the apparent motion of structures between the equivalent slices of the two subvolumes, $X_{z}^{\left(\text{even}\right)}$ and $X_{z}^{\left(\text{odd}\right)}$, where *z* denotes the slice index. The OF can be estimated assuming that the intensity of the pixels that represent those objects remains approximately constant:


(1) $$X_{z}^{\left(\text{odd}\right)}[y+v,x+u]\approx X_{z}^{\left(\text{even}\right)}[y,x],$$

where each coordinate (y,x) maps an area of the image $X_{z}^{\left(\text{even}\right)}$ to an area of the image $X_{z}^{\left(\text{odd}\right)}$ through the corresponding displacement vector (v,u). This allows us to build a projection to spatially bring the former to the latter image:


(2) $$\overset{\left(\text{even}\to \text{odd}\right)}{d_{z}}(X_{z}^{\left(\text{even}\right)})=X_{z}^{\left(\text{even}\to \text{odd}\right)}\approx X_{z}^{\left(\text{odd}\right)},$$

where $\overset{\left(\text{even}\to \text{odd}\right)}{d_{z}}=\{(v,u)\}$ is the displacement vector field. The same applies in the reverse direction:


(3) $$X_{z}^{\left(\text{even}\right)}[y+v,x+u]\approx X_{z}^{\left(\text{odd}\right)}[y,x],$$

(4) $$\overset{\left(\text{odd}\to \text{even}\right)}{d_{z}}(X_{z}^{\left(\text{odd}\right)})=X_{z}^{\left(\text{odd}\to \text{even}\right)}\approx X_{z}^{\left(\text{even}\right)}.$$

The OF problem therefore consists of estimating the displacement vector field d given the intensity constancy assumption (Equations 1 and 3) and under the constraint that d must be smooth. Here we use the Farnebäck algorithm (Farnebäck 2003), which minimizes the local Mean Square Error between the projection $X_{z}^{\left(\text{even}\to \text{odd}\right)}$ and its corresponding reference slice $X_{z}^{\left(\text{odd}\right)}$ (Equation 2), and the projection $X_{z}^{\left(\text{odd}\to \text{even}\right)}$ and its reference $X_{z}^{\left(\text{even}\right)}$ (Equation 4), using a sliding window that models the neighborhood with quadratic polynomials. The size *w* of this window controls the trade-off between estimation accuracy and robustness against noise. Another key parameter is the number of levels *l* of a Gaussian pyramid, which accounts for large displacements.

After OF estimation, the motion is corrected for by warping (i.e. non-rigidly registering) every slice of each subvolume towards its corresponding slice in the complementary subvolume, thereby generating the registered subvolumes:


(5) $$X^{\left(\text{even}\to \text{odd}\right)}=\{X_{z}^{\left(\text{even}\to \text{odd}\right)}\}=\{\overset{\left(\text{even}\to \text{odd}\right)}{d_{z}}(X_{z}^{\left(\text{even}\right)})\},$$

(6) $$X^{\left(\text{odd}\to \text{even}\right)}=\{X_{z}^{\left(\text{odd}\to \text{even}\right)}\}=\{\overset{\left(\text{odd}\to \text{even}\right)}{d_{z}}(X_{z}^{\left(\text{odd}\right)})\}.$$

This enables the generation of registered input-target pairs with enhanced structural consistency while preserving noise independence: $\left(X^{\left(\text{even}\right)},X^{\left(\text{odd}\to \text{even}\right)}\right)$ and $\left(X^{\left(\text{even}\to \text{odd}\right)},X^{\left(\text{odd}\right)}\right)$ (indicated by the turquoise and cyan arrows, respectively, in Fig. 1). Similar to practices in cryo-ET (Buchholz *et al.*  2019a, b), the subvolumes are divided into 3D patches that are randomly sampled to form the input-target pairs used to train the U-Net architecture underlying the N2N denoising model (Fig. 1). Once trained, the model is applied to the original noisy volume X, operating on a 3D patch-by-patch basis, to yield the final denoised volume Y (see the inference branch in Fig. 1).

## 3 Results

### 3.1 Datasets and parameters

A set of datasets representative of different samples (cells and tissues), preparation and imaging conditions (chemical fixation, high-pressure freezing and freeze substitution [HPF/FS], cryo), instruments, and slice thicknesses (5–50 nm) was selected to evaluate the method (Table 1).

**Table 1:** Datasets.

ID	Reference	Sample	Condition	Instrument	Pixel size	Slice thickness
**1**	Knott *et al.* (2011) ^a^	Brain tissue	chem.fix	Zeiss NVision 40	5 nm	5 nm
**2**	Hennies *et al.* (2020) ^b^	HeLa cell	HPF/FS	Zeiss Crossbeam 540	5 nm	8 nm
**3**	Martin-Solana *et al.* (2024)	Brain tissue	HPF/FS	Zeiss Crossbeam 550	5 nm	15 nm
**4**	Kelley *et al.* (2026) ^c^	Chlamy. cell	cryo	Thermo Sci. Hydra V	5 nm	20 nm
**5**	Dumoux *et al.* (2023) ^d^	Brain tissue	cryo	Thermo Sci. Hydra V	6.7 nm	50 nm
**6**	Dumoux *et al.* (2023) ^e^	HeLa cell	cryo	Thermo Sci. Hydra V	6.7 nm	50 nm

a https://www.epfl.ch/labs/cvlab/data/data-em.

b-e https://www.ebi.ac.uk/EMPIAR, IDs: ^b^10311, ^c^11275, ^d^11415, ^e^11419.

The actual implementation of N2N-EO and N2N-REO relies on cryoCARE N2N (Buchholz *et al.* 2019a) and non-rigid registration using the Farnebäck optical flow algorithm available in OpenCV (pseudocode in Figs S1 and S2, available as supplementary data at *Bioinformatics* online). For the N2N training, the default parameters were employed (a U-Net of depth 3, kernel size 3, 16 initial filters, batch size 16, and 50 epochs) with cubic 3D patches of 16^3^ voxels. For the OF, the key parameters, window size and number of levels were set to their optimal values of 5 and 3, respectively, as determined in a previous study (González-Ruiz *et al.* 2023).

### 3.2 Metrics for objective evaluation

Complementary to the visual analysis, we used a selected set of metrics for an objective evaluation to demonstrate the superior performance of N2N-REO. The first metric is based on the single-image Fourier Ring Correlation (FRC), also known as Self-FRC (Koho *et al.* 2019, Fernandez *et al.* 2020). Here, every slice of the volume is decomposed into subimages of half the original size by taking every other pixel along both spatial dimensions. The 2×-subsampled subimages are then subjected to FRC to yield the correlation of the slice content as a function of the spatial frequency. This metric accounts for both SNR and blurring (lower SNR or greater blur results in a lower FRC at the corresponding spatial frequency), thereby enabling the assessment of the effective resolution of each slice. In this work, the average Self-FRC computed over all slices is utilized as the final metric, referred to as ASFRC.

Furthermore, we also incorporated well-known similarity metrics from the computer vision field: the Structural Similarity Index Measure (SSIM), the Pearson’s Correlation Coefficient (PCC) and the Peak SNR (PSNR) (Wang *et al.* 2004, Szeliski 2022). These metrics were computed between each denoised volume and its corresponding original noisy volume to assess the preservation of the original signal upon denoising (with larger values indicating better preservation). The combination of these metrics with ASFRC aims to evaluate the robustness against blurring and the structure-preserving capability of our method.

### 3.3 Influence of the slice thickness

To study the effect of the slice thickness on the performance of the denoising methods, a series of datasets with slice thicknesses of 5, 10, 20, 30, and 40 nm was synthesized from Dataset 1 (slice thickness 5 nm) by omitting the corresponding number of intermediate slices. These generated datasets were then subjected to denoising using both N2N-EO and N2N-REO. Figure 2 shows a selected slice present in all denoised volumes (the 5 nm case not shown for conciseness). N2N-REO (Fig. 2B) exhibits noise reduction with good structure preservation regardless of the slice thickness. In contrast, N2N-EO shows a clear susceptibility to blurring, particularly severe at 30 and 40 nm (Fig. 2C and Fig. S3, available as supplementary data at *Bioinformatics* online). This behavior is especially noticeable in mitochondria cristae, vesicles, and membranous areas in general. Therefore, as indicated by the divergence between N2N-REO and N2N-EO, motion correction becomes paramount beyond 10 nm, growing increasingly critical as thickness increases.

**Figure 2:**
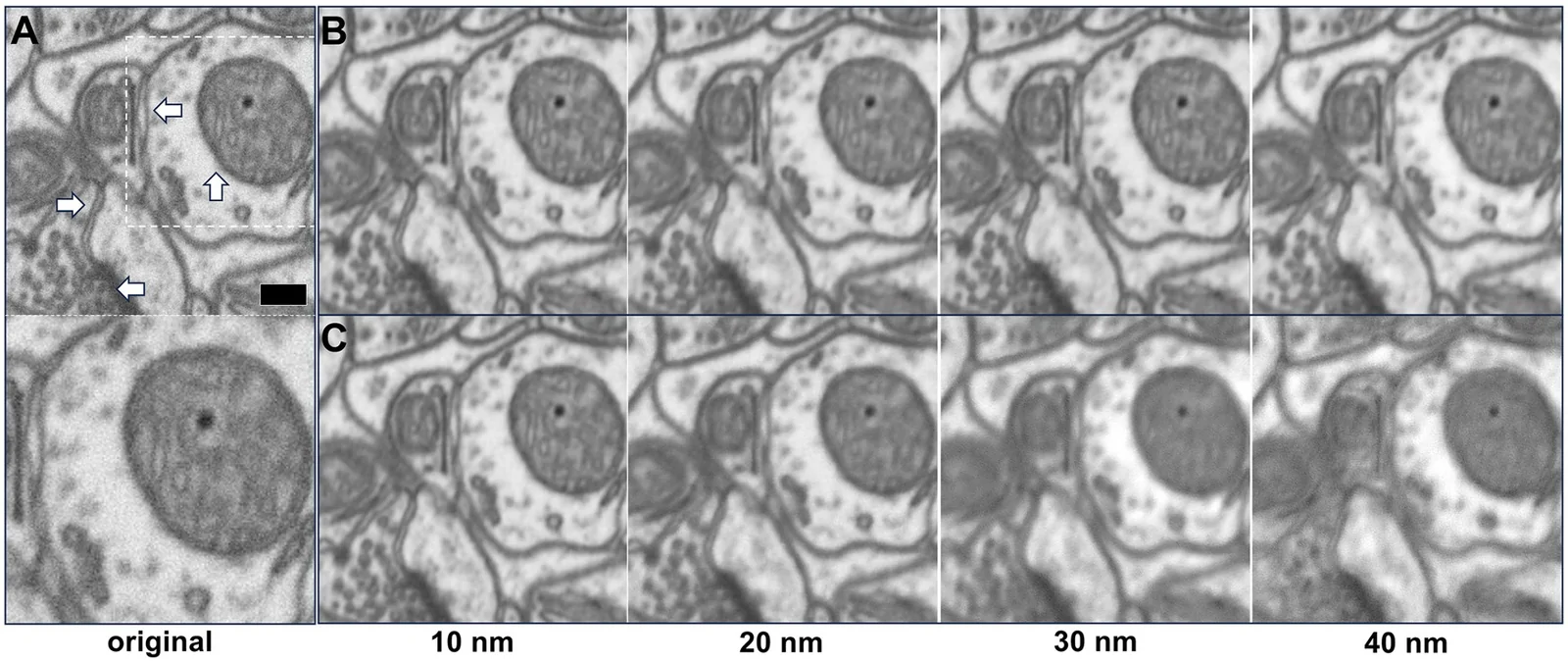
Performance of motion-corrected N2N as a function of slice thickness. (A) Original volume. The dashed box is zoomed-in in the bottom panel. Some areas of interest (membranes, vesicles, and mitochondria cristae) are marked with arrows. (B) Results of N2N-REO (motion correction) for volumes with slice thicknesses of 10 to 40 nm (left to right). (C) Equivalent results of N2N-EO (no motion correction). Bar: 200 nm.

To further substantiate the performance of the methods at different slice thicknesses, we performed a quantitative analysis by computing objective metrics from the volumes. First, ASFRC curves were obtained for all volumes to estimate the overall cleanliness and structural sharpness. Figure 3 shows that N2N-REO consistently outperforms N2N-EO at all spatial frequencies, with the performance gap becoming particularly pronounced for slice thicknesses >10 nm. Furthermore, N2N-EO degrades significantly at large values (30 and 40 nm). These results indicate that N2N-REO provides denoised volumes that retain information at higher resolution. Second, SSIM, PCC, and PSNR metrics were calculated to assess the preservation of the original signal (Table S1, available as supplementary data at *Bioinformatics* online). The ratio N2N-REO/N2N-EO for all metrics (Fig. 4) confirms the superiority of N2N-REO, exhibiting a consistent upward trend for slice thicknesses exceeding 10 nm.

**Figure 3:**
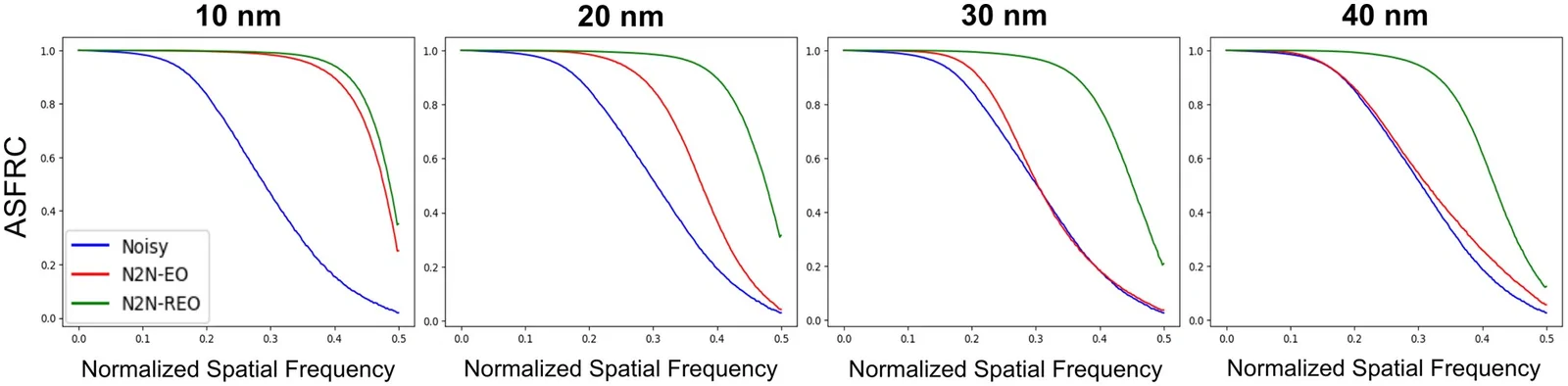
Average Self Fourier Ring Correlation (ASFRC) as a function of slice thickness. From left to right, ASFRC curves for slice thicknesses of 10, 20, 30, and 40 nm. Colors: original noisy volume (blue), N2N-EO (red), N2N-REO (green). On the X axis, the maximum normalized spatial frequency (0.5) corresponds to that from the 2×-subsampled subimages used for the ASFRC calculation.

**Figure 4:**
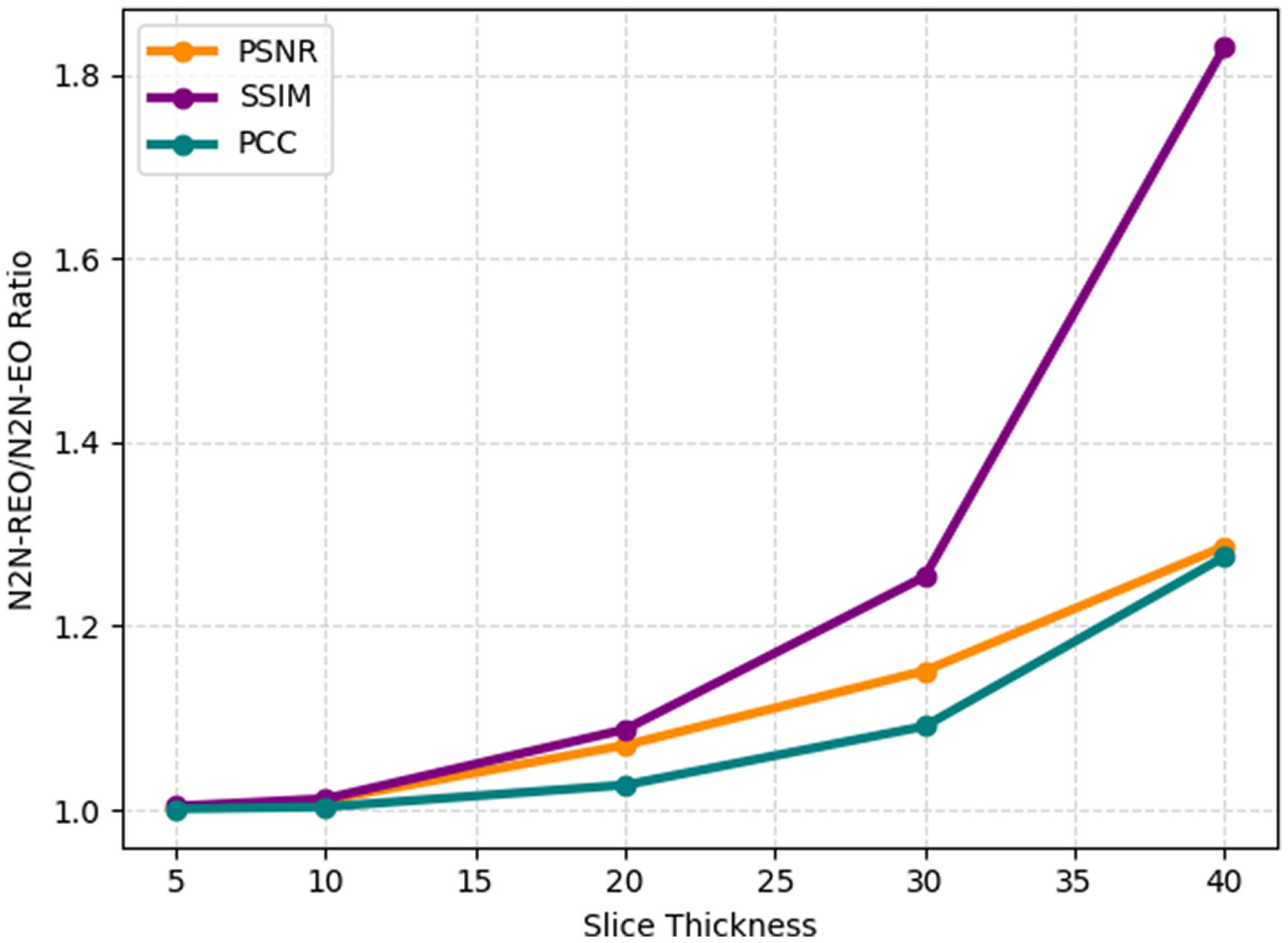
Metrics PSNR, SSIM, and PCC as a function of slice thickness. The metrics are presented as a ratio N2N-REO/N2N-EO.

To provide an additional independent evaluation, we conducted an experiment using synthetic phantoms with a clean reference and quantitative metrics. The results align with the findings in this section and confirm the superiority of N2N-REO (see Section S3, available as supplementary data at *Bioinformatics* online).

N2N relies on the strict assumption that the noise in the training pairs is independent. We conducted an experiment confirming that the warping applied in N2N-REO does not violate this requirement (Section S4, available as supplementary data at *Bioinformatics* online).

### 3.4 Evaluation with experimental data

We then applied the methods to the experimental datasets listed in Table 1. The results for Dataset 1 (slice thickness 5 nm) were visually indistinguishable from those presented in Fig. 2 (panels labeled as 10 nm), showing a sharp signal on a clean volume, with no discernible difference between N2N-REO and N2N-EO.

Figure 5 presents the results for the two datasets prepared with HPF/FS. In both cases, the structural signal is preserved, noise is effectively suppressed, and, particularly in the second case, the background is substantially flattened. In accordance with the findings in the previous section, N2N-REO and N2N-EO display negligible differences for Dataset 2 (slice thickness 8 nm) whereas for Dataset 3 (15 nm) N2N-REO outperforms N2N-EO, as the latter exhibits blurring that is particularly noticeable in membranous regions.

**Figure 5:**
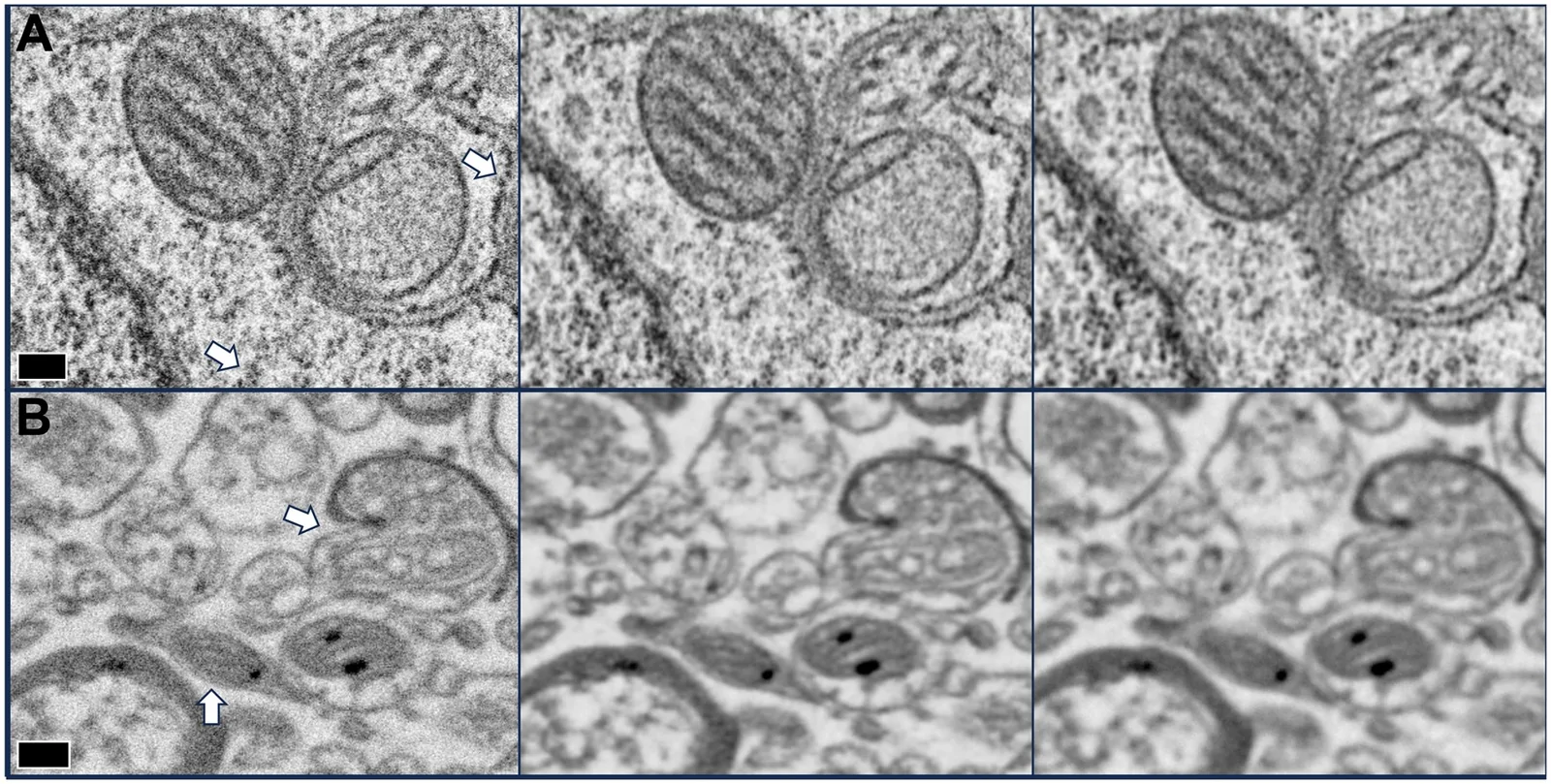
Denoising FIB/SEM datasets prepared with HPF/FS. From left to right, a slice of the original volume, denoised with N2N-REO (motion correction) and with N2N-EO (no motion correction). (A) HeLa cell (Dataset 2). (B) Brain tissue (Dataset 3). Some areas with more evident effects are marked (arrows). Bar: 200 nm.

Finally, we evaluated the methods with the three datasets under cryo conditions. Cryo-FIB/SEM of vitrified samples is in general characterized by extremely low contrast, low SNR conditions, and slice thicknesses typically exceeding 20 nm, thus representing challenging case studies. As presented in Fig. 6 and Figs S4 and S5, available as supplementary data at *Bioinformatics* online, N2N-REO (motion correction) succeeds in significantly reducing the noise and flattening the background while demonstrating outstanding abilities to preserve sharpness in the structures. In contrast, N2N-EO (no motion correction) undergoes substantial overall blurring, leaving certain membranous constituents (e.g. Golgi stacked cisternae and mitochondria cristae) barely perceptible in some areas. These results are consistent with the large slice thickness inherent to cryo-FIB/SEM and corroborate the paramount importance of motion correction in N2N denoising.

**Figure 6:**
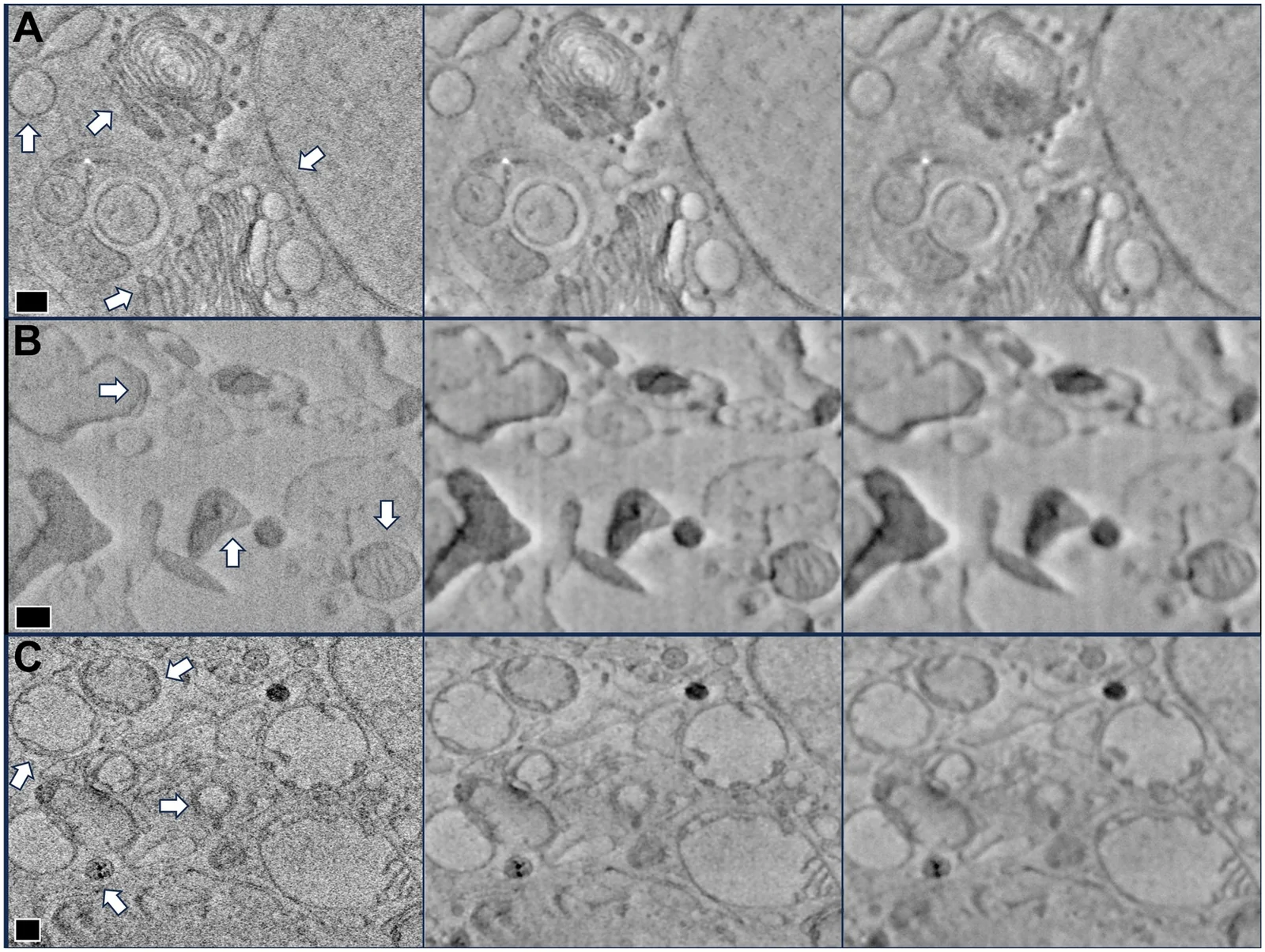
Denoising datasets from vitrified samples acquired with cryo-FIB/SEM. From left to right, a slice of the original volume, denoised with N2N-REO (motion correction) and with N2N-EO (no motion correction). (A) Chlamydomonas cell (Dataset 4). (B) Brain tissue (Dataset 5). (C) HeLa cell (Dataset 6). Some membranous areas exhibiting more evident effects are marked (arrows). Bar: 200 nm.

To quantitatively evaluate the quality of the denoised volumes, the objective metrics were calculated. The ASFRC curves (Fig. 7) show that N2N-REO is superior to N2N-EO at all spatial frequencies in all cases. ASFRC thus confirms that N2N-REO yields denoised volumes with details of higher resolution and avoids blurring compared to N2N-EO. Consistent with the previous section, the performance gap in ASFRC between the two approaches becomes apparent beyond 10 nm and widens for larger slice thicknesses. Additionally, the SSIM, PCC and PSNR metrics (Table S2, available as supplementary data at *Bioinformatics* online) for N2N-REO are consistently higher than those for N2N-EO, further demonstrating its superior ability to preserve original structural details.

**Figure 7:**
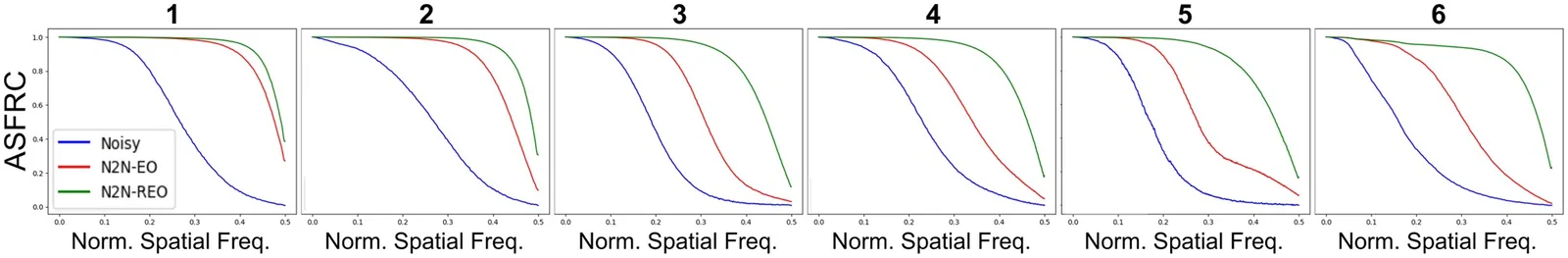
Average Self Fourier Ring Correlation (ASFRC) for the six datasets in Table 1. From left to right, the slice thickness of the datasets is in increasing order: 5, 8, 15, 20, 50, and 50 nm. Colors: original noisy volume (blue), N2N-EO (red), N2N-REO (green). The maximum value (0.5) on the X axis represents the maximum spatial frequency corresponding to the 2×-subsampled subimages used for the ASFRC calculation.

The mismatch in slice spacing between the training and inference stages (even/odd subvolumes with double spacing versus the original volume, respectively) might, in principle, introduce structural blurring and reduced axial resolution in N2N-REO. Nevertheless, our results suggest that these effects are not apparent, thereby validating the N2N-REO approach for structure-preserving denoising in cryo-FIB/SEM.

### 3.5 Comparison with state-of-the-art methods

Next, we aimed to compare our motion-corrected N2N approach with respect to standard and state-of-the-art denoising methods in (cryo-)FIB/SEM. To this end, the challenging Cryo-dataset 4 was selected and subjected to standard Gaussian filtering (GF), Structure-Preserving Gaussian denoising (SPGD) (González-Ruiz *et al.* 2023, Zhu *et al.* 2024) and Anisotropic Nonlinear Diffusion (AND) (Moreno *et al.* 2018, Kapteijn *et al.* 2022). Figure 8 presents the results for areas containing Golgi complexes, showing that all methods succeed in reducing the noise to a similar level. Nevertheless, the drawbacks of GF, SPGD and AND are evident (see areas denoted by arrows). GF blurs the structures, while SPGD and AND—which are known to reinforce membranous structures—are prone to creating artifactual patterns and still introduce some blur in specific areas. Overall, N2N-REO manages to reduce noise while naturally preserving structural features without generating artifacts.

**Figure 8:**
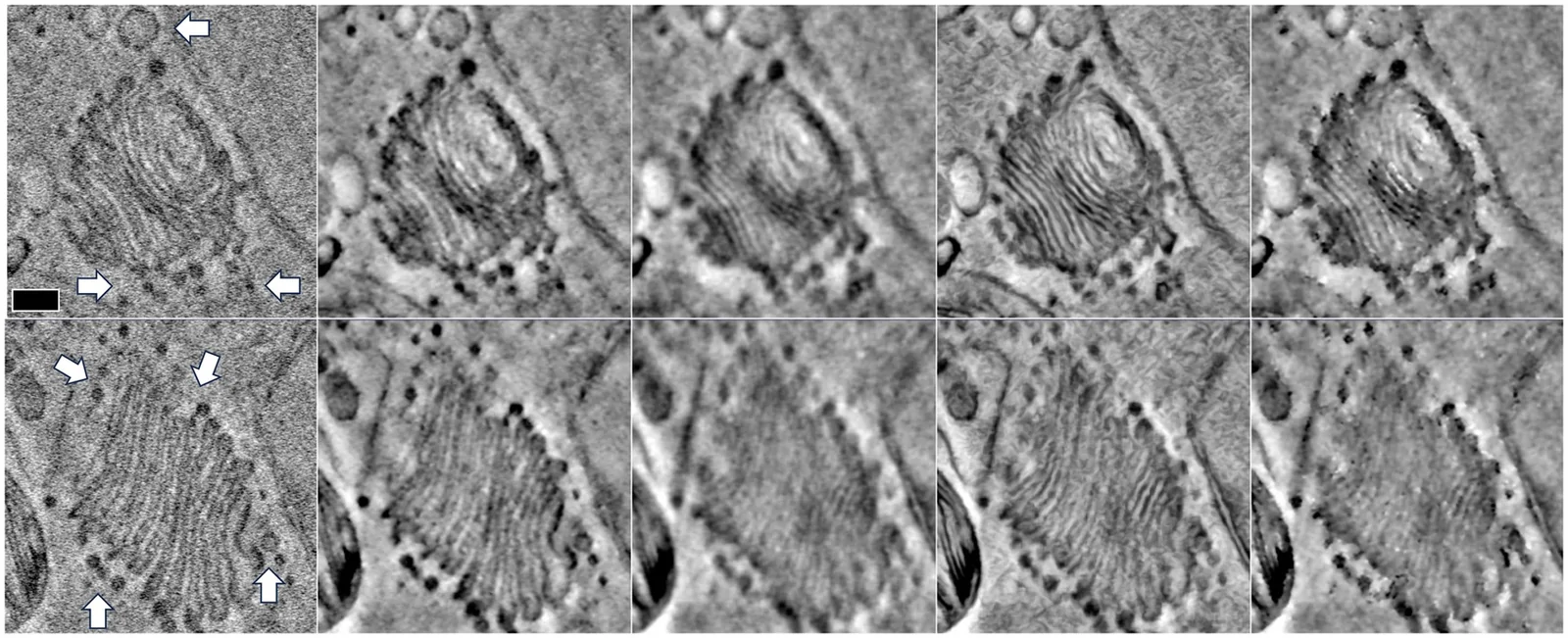
Comparison with other methods used in (cryo-)FIB/SEM. Selected areas of cryo-dataset 4. From left to right, a slice from the original volume and slices denoised using N2N-REO, Gaussian filtering, Structure-Preserving Gaussian denoising, and Anisotropic Nonlinear Diffusion. Areas susceptible to artifacts or blurring during denoising are marked with arrows. Bar: 200 nm.

### 3.6 Application to volume electron microscopy

(Cryo-)FIB/SEM represents a prominent modality within a suite of electron microscopy techniques, collectively known as volume electron microscopy (vEM), which enable the in-depth investigation of the 3D structure of cells and tissues (Collinson *et al.* 2023). Most vEM techniques rely on serial sectioning and imaging of the sample. Consequently, they could also benefit from our motion-corrected N2N denoising approach. To demonstrate this broader applicability, we applied N2N-REO to selected datasets from two vEM modalities. Figure 9 and Fig. S9, available as supplementary data at *Bioinformatics* online, illustrate the outstanding noise reduction, background flattening, and signal preservation achieved in both cases. Therefore, our approach can be used to facilitate interpretation of the volumes in vEM.

**Figure 9:**
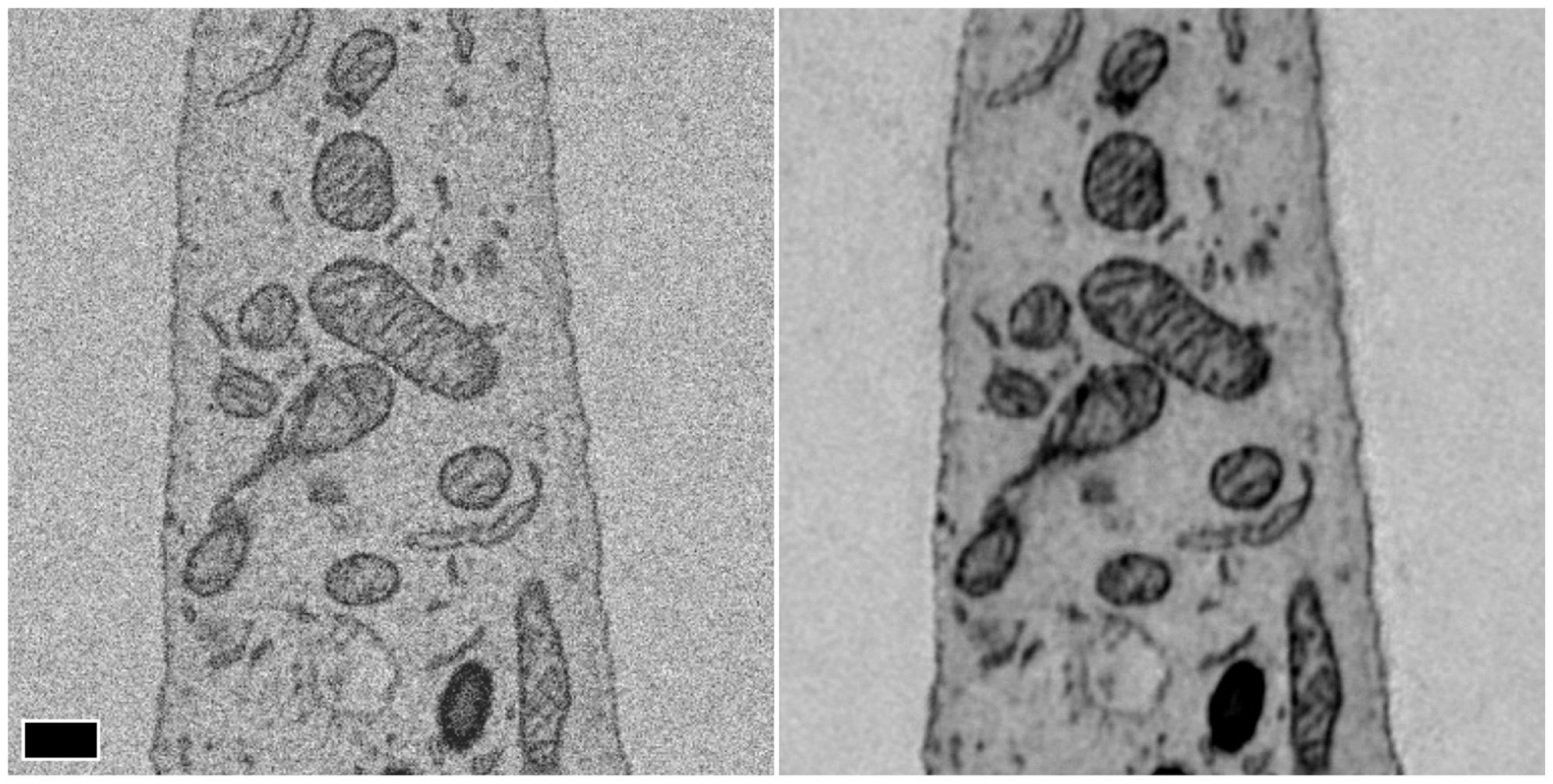
Motion-corrected N2N denoising applied to Array Tomography of chemically fixed cells (Nakamura *et al.* 2025) (EMPIAR, ID: 12 608) with a slice thickness of 50 nm. Bar: 500 nm.

## 4 Conclusion

We have introduced a self-supervised N2N approach to denoising single volumes in (cryo-)FIB/SEM. It splits the input volume into two subvolumes containing even/odd slices to conform the training pairs. The method includes a key motion-correction stage just prior to training, aiming at estimating and compensating for the structural discrepancies between the two subvolumes. Experimental results on (cryo-)FIB/SEM datasets demonstrate that the motion correction significantly improves the noise attenuation, structural preservation, and overall quality of the volumes, particularly for slice thicknesses larger than 10 nm. These findings suggest that data splitting combined with non-rigid registration provides an effective solution for self-supervised denoising in vEM scenarios where repeated acquisitions of the same volume are unfeasible.

## Supplementary Material

btag765_Supplementary_Data
