# Supplementary material for: Motion-corrected Noise2Noise denoising for structural analysis of cells and tissues with (cryo-)FIB/SEM
Source: Bioinformatics. 2026 Oct 16;42(10):btag765. doi: 10.1093/bioinformatics/btag765 (PMC13694117; doi:10.1093/bioinformatics/btag765)
Supplement: Supplementary file 1 [file btag765_supplementary_data.pdf]

Motion-corrected Noise2Noise denoising for structural analysis of cells and tissues with (cryo-)FIB/SEM

– Supplementary Material –

Vicente González-Ruiz and José-Jesús Fernández

Table of Contents

S1. Pseudo-code of the denoising methods.
S2. Evaluation with experimental data.
S3. Evaluation with simulated data.
S4. Evaluation of noise independence after warping.
S5. Denoising in other vEM modalities.

S1. Pseudo-code of the denoising methods.

```
# Routine for N2N-REO
N2N-REO(noisy_vol):
    # Extract Even Z-slices.
    even_vol = noisy_vol[0::2,:,:]
    # Extract Odd Z-slices.
    odd_vol  = noisy_vol[1::2,:,:]
    # Register even_vol to odd_vol.
    even_vol_registered = register_vol(even_vol, odd_vol)
    # Register odd_vol to even_vol.
    odd_vol_registered = register_vol(odd_vol, even_vol)
    # Train the N2N model.
    model = train(input =[even_vol,           even_vol_registered],
                  target=[odd_vol_registered, odd_vol            ])
    # Apply the trained model to denoise the volume.
    denoised_vol = predict(model, noisy_vol)
    # Return the denoised volume.
    return denoised_vol

# Routine to register a volume to a reference.
register_vol(vol_to_warp, reference_vol):
    registered_vol = empty_like(reference_vol)
    # Warp all slices individually.
    for z in range(reference_vol.shape[0]):
        registered_vol[z] = register_slice(vol_to_warp[z], reference_vol[z])
    # Return the registered volume.
    return registered_vol

# Routine to register a slice to a reference.
register_slice(slice_to_warp, reference_slice):
    # Estimate the motion with optical flow (OF).
    optical_flow = OF_estimator.estimate_2D_dense
                     (to_warp=slice_to_warp, target=reference_slice)
    # Warp the slice according to OF.
    warped_slice = Warper.project_2D(slice=slice_to_warp, flow=optical_flow)
    # Return the warped slice.
    return warped_slice
```

Figure S1: Pseudo-code of the N2N-REO approach. Training is carried out by applying the following input-target pairs $(\boldsymbol{X}^{(\text{even})}, \boldsymbol{X}^{(\text{odd}\rightarrow\text{even})})$ and $(\boldsymbol{X}^{(\text{even}\rightarrow\text{odd})}, \boldsymbol{X}^{(\text{odd})})$. Here, $\boldsymbol{X}^{(\text{even})}$ and $\boldsymbol{X}^{(\text{odd})}$ are the subvolumes containing the even and odd slices, respectively (i.e. `even_vol` and `odd_vol` in the pseudocode), the warped version of $\boldsymbol{X}^{(\text{even})}$ to $\boldsymbol{X}^{(\text{odd})}$ is $\boldsymbol{X}^{(\text{even}\rightarrow\text{odd})}$ (i.e. `even_vol_registered` in the pseudocode) and the warped version of $\boldsymbol{X}^{(\text{odd})}$ to $\boldsymbol{X}^{(\text{even})}$ is $\boldsymbol{X}^{(\text{odd}\rightarrow\text{even})}$ (i.e. `odd_vol_registered` in the pseudocode) .

```
# Routine for N2N-EO
N2N-EO(noisy_vol):
    # Extract Even Z-slices.
    even_vol = noisy_vol[0::2,:,:]
    # Extract Odd Z-slices.
    odd_vol  = noisy_vol[1::2,:,:]
    # Train the N2N model.
    model = train(input=[even_vol], target=[odd_vol])
    # Apply the trained model to denoise the volume.
    denoised_vol = predict(model, noisy_vol)
    # Return the denoised volume.
    return denoised_vol
```

Figure S2: Pseudo-code of the N2N-EO approach. Training is carried out by applying the subvolumes containing the even and odd slices as input and target, respectively.

S2. Evaluation with experimental data

	SSIM		PCC		PSNR	
slice thickness	N2N-EO	N2N-REO	N2N-EO	N2N-REO	N2N-EO	N2N-REO
5 nm	0.79	0.79	0.96	0.96	27.39	27.45
10 nm	0.84	0.85	0.96	0.96	28.12	28.41
20 nm	0.81	0.88	0.93	0.95	26.02	27.85
30 nm	0.70	0.87	0.87	0.95	23.52	27.08
40 nm	0.48	0.87	0.74	0.94	19.73	25.39

Table S1: SSIM, PCC and PSNR comparing N2N-REO and N2N-EO for the synthetic datasets created from experimental Dataset 1 with different slice thickness (Section 3.3 of the main text).

	SSIM		PCC		PSNR	
dataset	N2N-EO	N2N-REO	N2N-EO	N2N-REO	N2N-EO	N2N-REO
1	0.79	0.79	0.96	0.96	27.39	27.45
2	0.59	0.62	0.79	0.80	17.86	18.16
3	0.56	0.62	0.84	0.86	26.01	26.61
4	0.54	0.62	0.59	0.70	29.05	29.91
5	0.30	0.40	0.53	0.62	23.42	24.06
6	0.42	0.50	0.55	0.64	27.40	28.07

Table S2: SSIM, PCC and PSNR comparing N2N-REO and N2N-EO for the experimental datasets listed in Table 1.

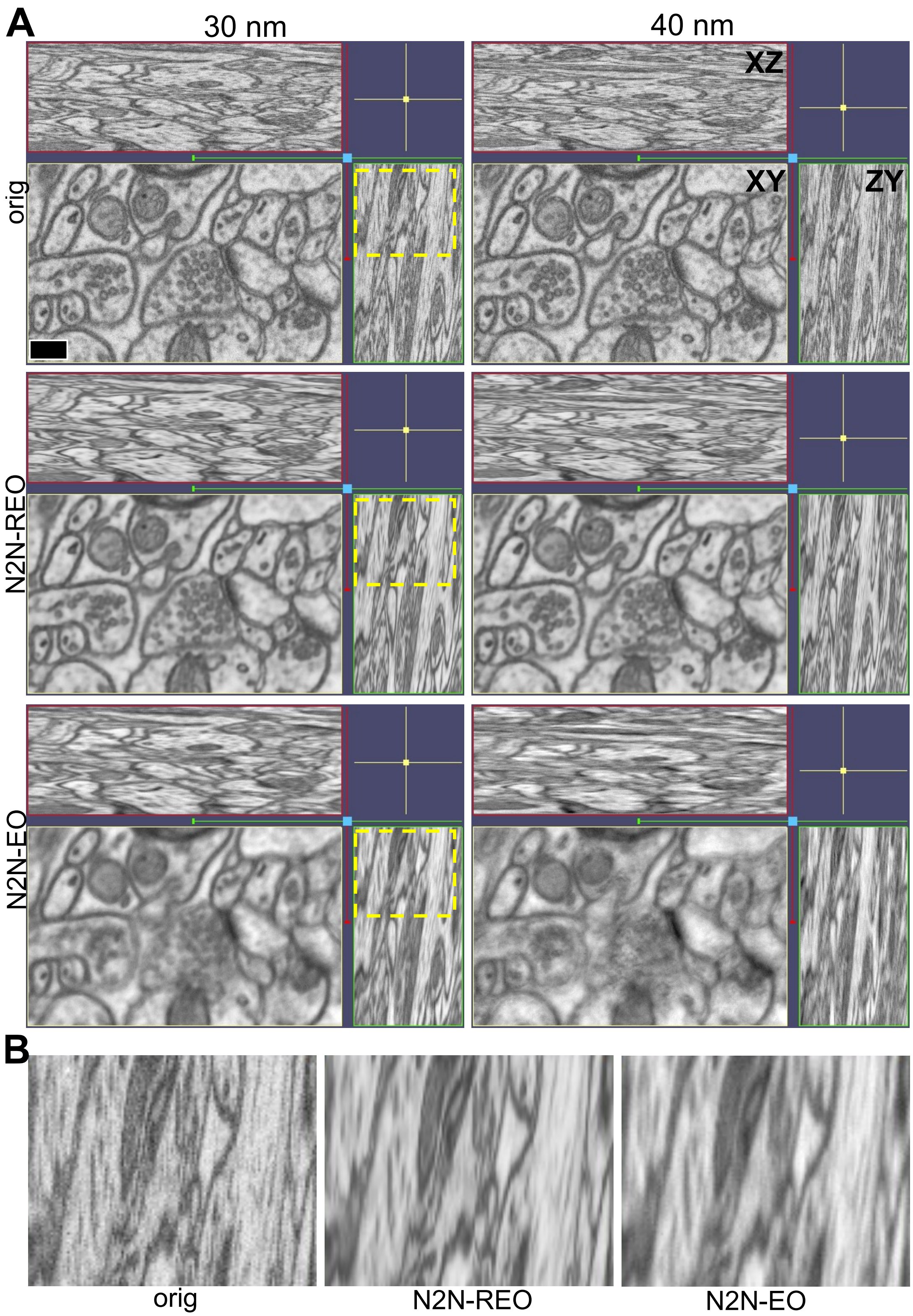

Figure S3: Denoising performance on the Dataset 1 with synthetic slice thicknesses of 30 and 40 nm. Selected orthogonal slices XY, XZ, YZ from the original volume and denoised with N2N-REO and N2N-EO are shown in (A). Areas marked with yellow boxes are magnified in (B). N2N-REO provides a cleaner solution with sharper definition of the structural features whereas N2N-EO undergoes severe blurring, particularly noticeable in mitochondria, vesicles and membranes. Bar: 200 nm.

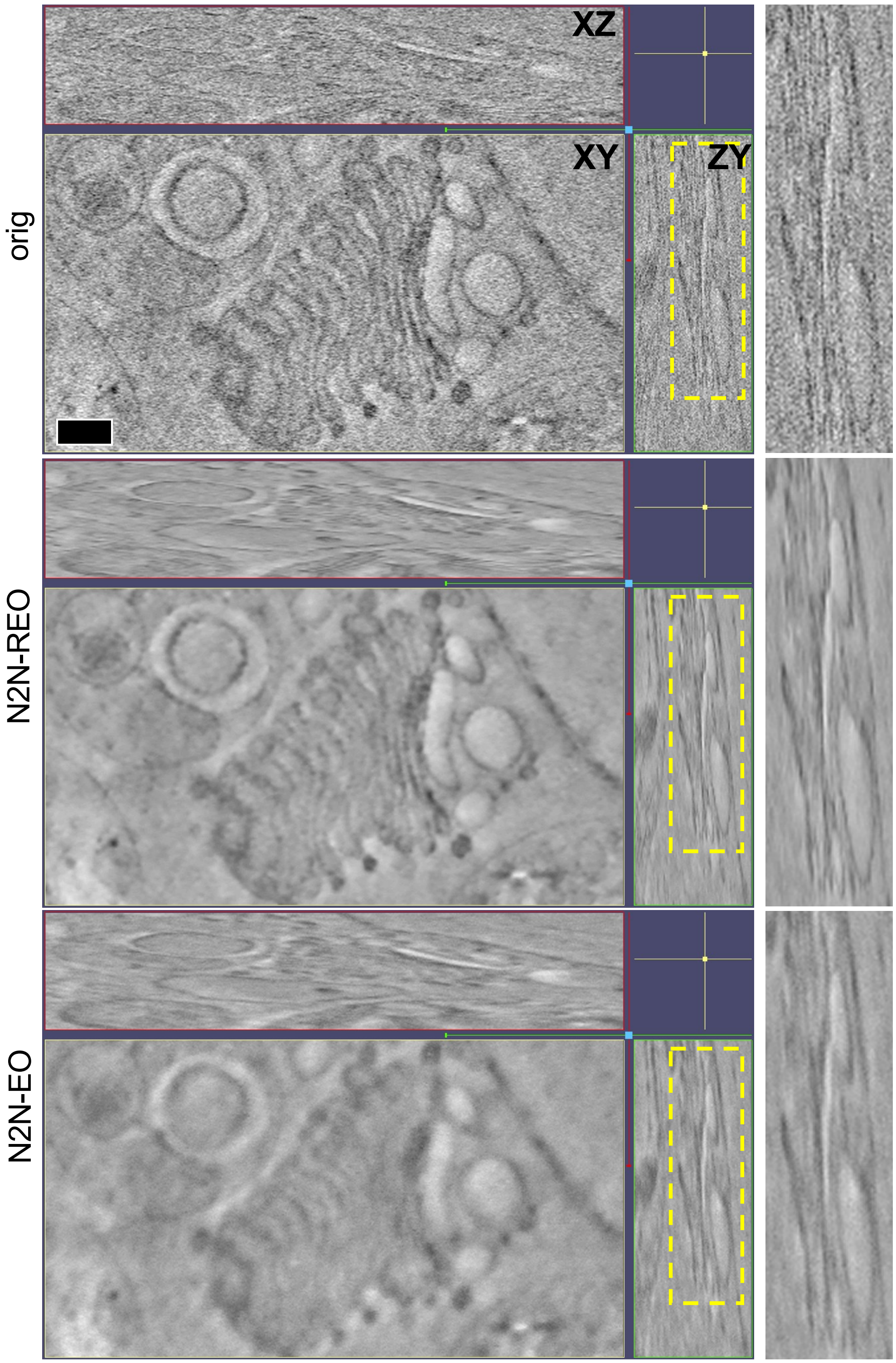

Figure S4: Denoising dataset from a vitrified Chlamydomonas cell (Dataset 4) acquired with cryo-FIB/SEM using a slice thickness of 20 nm. Selected orthogonal slices XY, XZ, YZ from the original volume and denoised with N2N-REO and N2N-EO are shown. Areas marked with yellow boxes are magnified in the rightmost panels. N2N-REO provides a remarkably clean and sharp solution with a flattened background compared to the original volume, visible across all orthogonal planes. However, N2N-EO is severely affected by blurring. Bar: 200 nm.

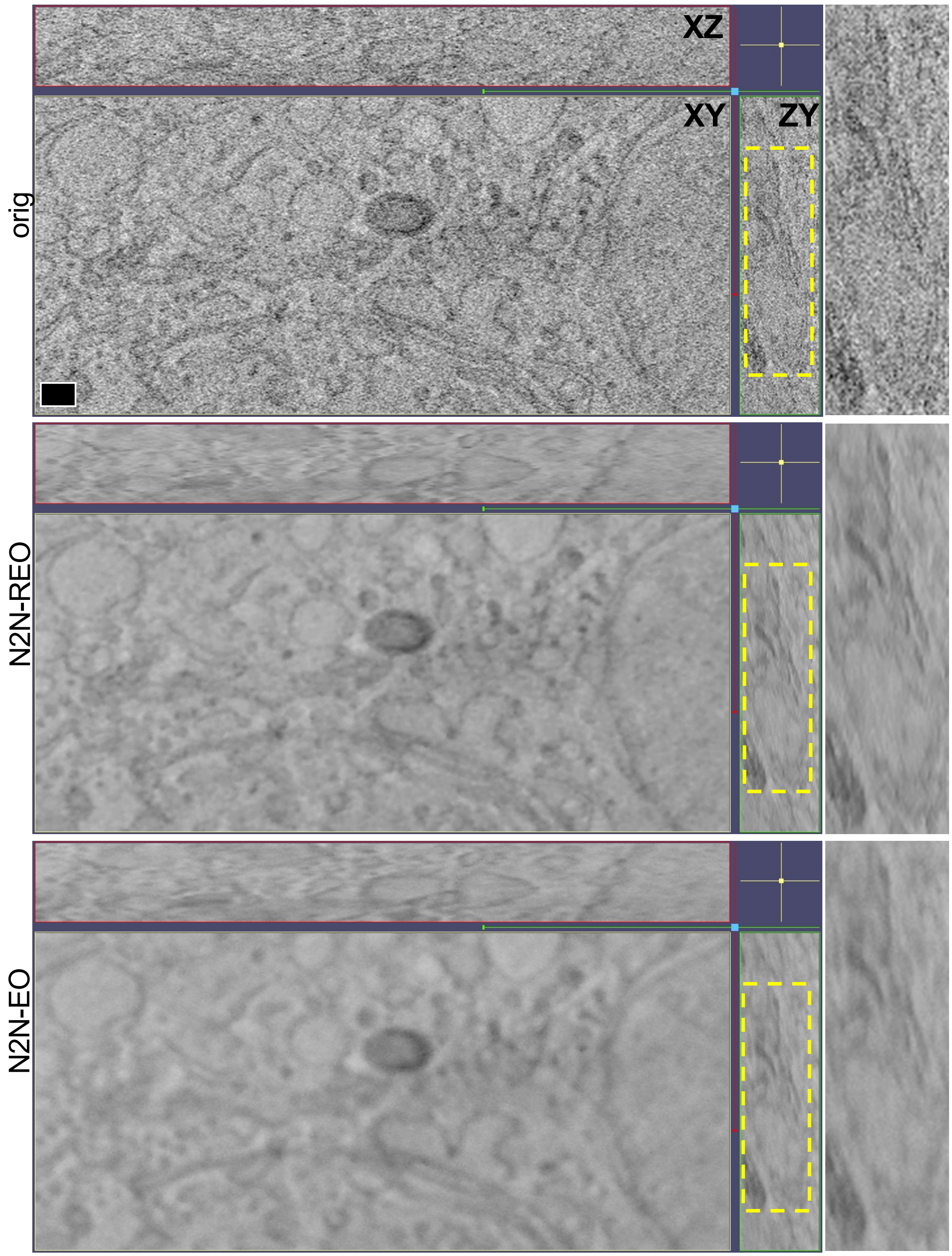

Figure S5: Denoising dataset from a vitrified HeLa cell (Dataset 6) acquired with cryo-FIB/SEM using a slice thickness of 50 nm. Selected orthogonal slices XY, XZ, YZ from the original volume and denoised with N2N-REO and N2N-EO are shown. Areas marked with yellow boxes are magnified in the rightmost panels. N2N-REO provides a remarkably clean and sharp solution compared to the original volume, visible across all orthogonal planes. However, N2N-EO is severely affected by blurring, particularly noticeable in membranes. Bar: 200 nm.

S3. Evaluation with simulated data.

To provide an additional, independent evaluation, we conducted an experiment with synthetic data. A synthetic FIB/SEM phantom of the cytoplasm of a human macrophage was constructed based on segmentation data available in the EMPIAR database, ID: 13420 [1] (Szkalisity *et al.* (2025) EMBO J. 44:3, doi: https://doi.org/10.1038/s44318-025-00423-2). The phantom contained mitochondria (gray), endoplasmic reticulum (dark gray) and lipid droplets (black) on a white cytoplasm (Fig. **S6**). The original data had a pixel size of 6 nm and a slice thickness of 12 nm. A series of phantoms with the same pixel size and slice thickness of 12, 24, 36 and 48 nm was then built to serve as references. Gaussian noise was then added to yield a signal-to-noise ratio (SNR) of 0.1, simulating conditions close to those typically encountered in cryo-imaging.

The noisy phantoms were denoised using N2N-REO and N2N-EO. Fig. **S6** illustrates the results for the specific phantom with a slice thickness of 36 nm. Here, the susceptibility to blurring of N2N-EO is clearly observed. In contrast, N2N-REO provide sharp denoised outputs with good signal preservation and well-defined organelle boundaries. Similar performance was observed with the phantoms with other slice thicknesses (12, 24 and 48 nm).

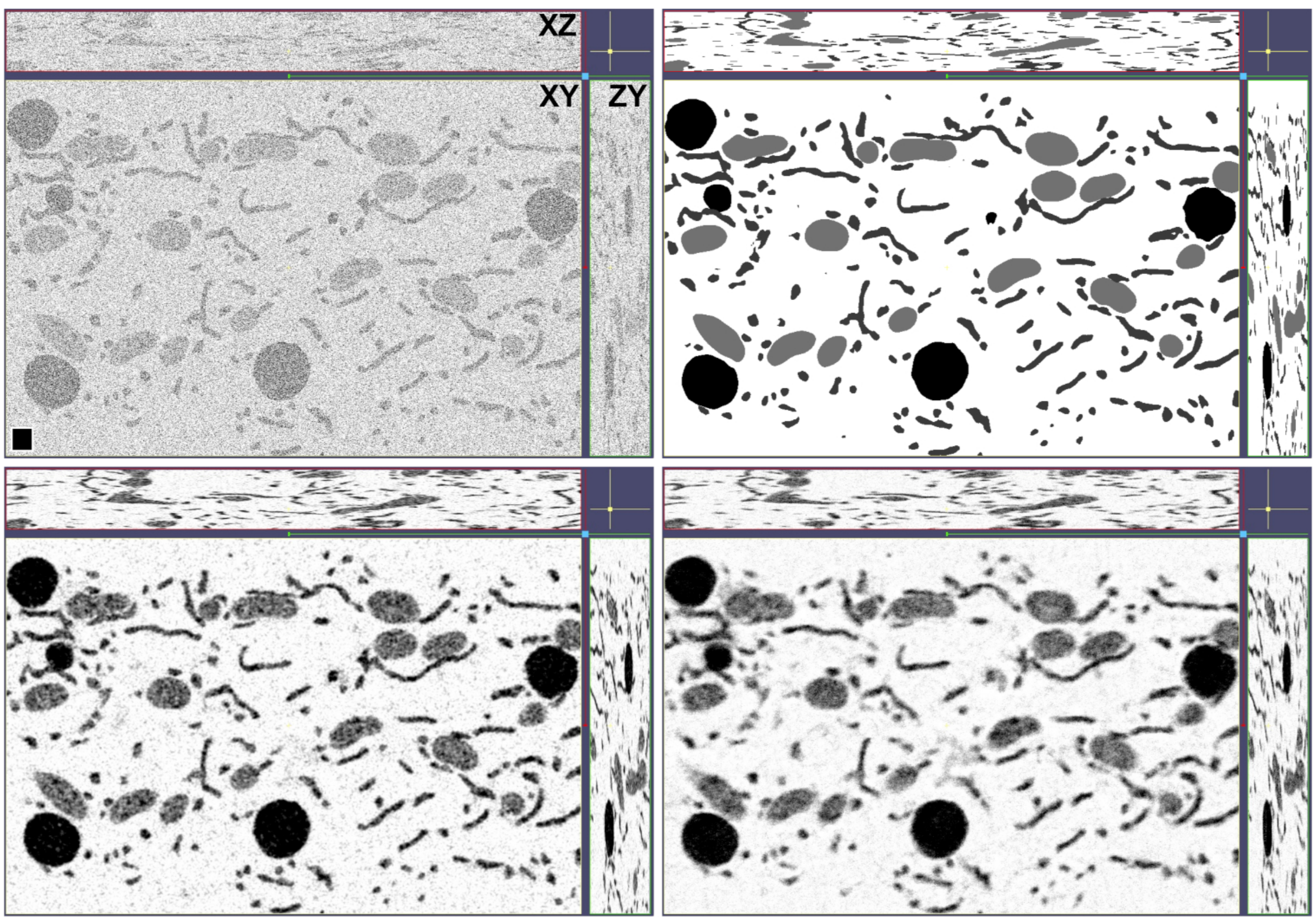

Figure S6: Visual evaluation of denoising performance on the synthetic phantom with a slice thickness of 36 nm. From left to right and top to bottom: noisy phantom (SNR=0.1), clean reference, denosing results from N2N-REO and N2N-EO. Selected XY, XZ and ZY orthogonal planes are shown. The phantom contains mitochondria (gray), endoplasmic reticulum (dark gray) and lipid droplets (black) on a white cytoplasm. N2N-REO yields a sharp denoised volume that resembles the clean reference, whereas N2N-EO provides a volume severely affected by blurring. Bar: 200 nm.

[1] https://www.ebi.ac.uk/empiar/EMPIAR-13420/

To objectively evaluate denoising quality, the SSIM, PCC and PSNR metrics (Table **S3**) as well as the Fourier Ring Correlation (FRC) curves (Fig. **S7**) were computed between the denoised volumes and the corresponding clean references. Table **S3** clearly demonstrates the superiority of N2N-REO across all cases in terms of SSIM, PCC and PSNR.

The FRC (Harauz & van Heel (1986). Optik 73:146–156) measures the normalized cross-correlation coefficient between two images over corresponding concentric rings of increasing radius in Fourier space (i.e. as a function of spatial frequency). Here, the FRC curves were calculated slice-by-slice and averaged across the entire volume to generate the plots presented in Fig. **S7**. These curves indicate that N2N-REO consistently outperforms N2N-EO, achieving a substantial boost in spatial resolution. Conversely, N2N-EO performs poorly, with its FRC curves dropping near zero at spatial frequencies between 0.1 and 0.2, thereby quantitatively reflecting its tendency to blurring. The performance gap between N2N-REO and N2N-EO becomes larger with greater slice thickness. Overall, these findings on simulated data align with the experimental results reported in the main text and confirm the superior performance of N2N-REO.

	SSIM		PCC		PSNR	
slice thickness	N2N-EO	N2N-REO	N2N-EO	N2N-REO	N2N-EO	N2N-REO
12 nm	0.30	0.34	0.90	0.93	17.86	18.96
24 nm	0.31	0.37	0.87	0.91	16.54	17.97
36 nm	0.30	0.35	0.83	0.89	15.82	17.25
48 nm	0.32	0.37	0.81	0.87	15.69	17.05

Table S3: SSIM, PCC and PSNR comparing N2N-REO and N2N-EO for the synthetic phantoms with different slice thickness.

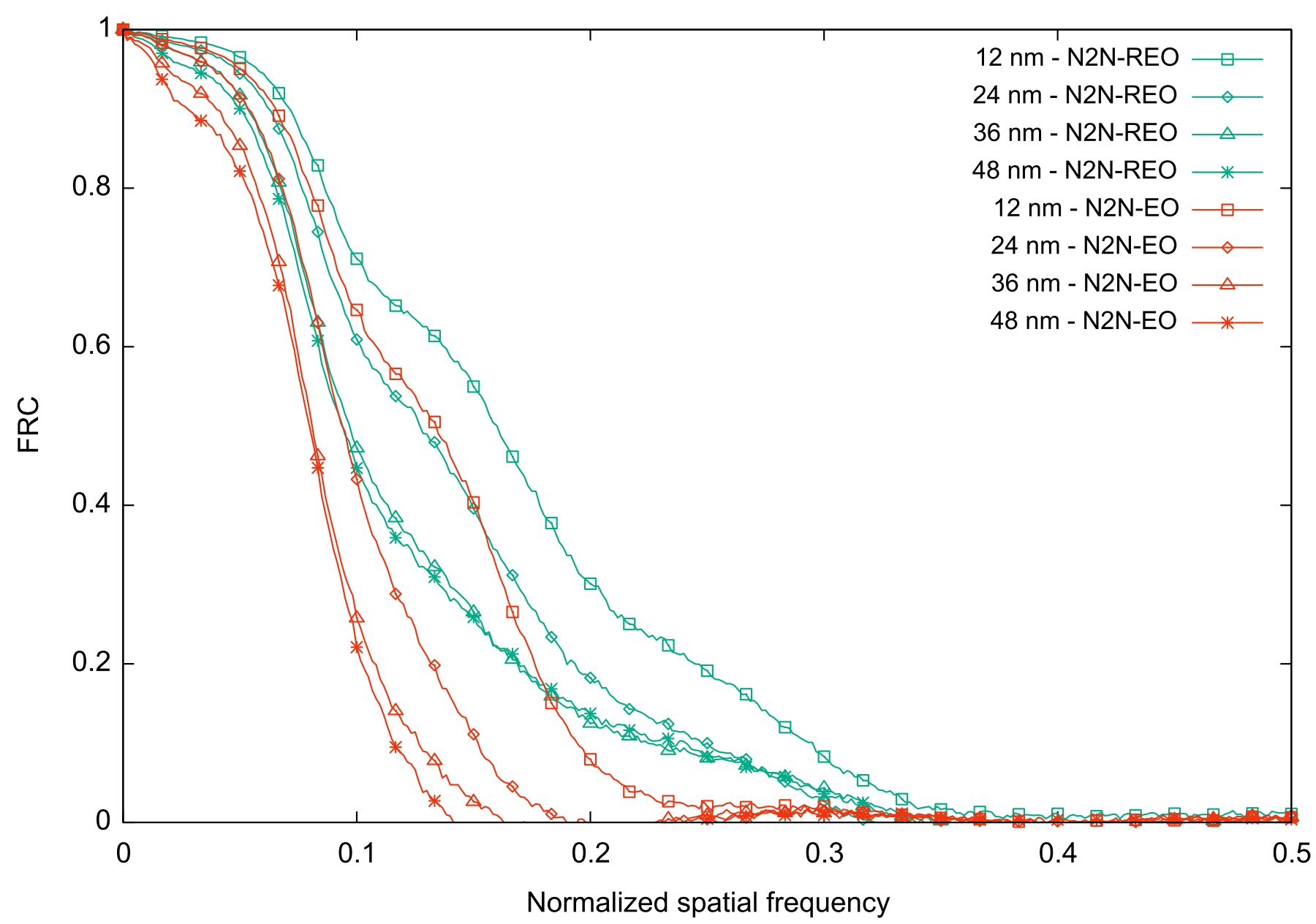

Figure S7: Fourier Ring Correlation curves obtained between the denoised volumes and the corresponding synthetic clean phantoms. Each of these curves were obtained by computing the FRC slice-by-slice and calculating the average across the entire volume.

S4. Evaluation of noise independence after warping.

Noise2Noise relies on the strict assumption that noise in the training image pairs is independent. The warping process in N2N-REO raises the question of whether non-rigid registration introduces correlated noise, thereby violating this core requirement. To evaluate noise independence after image warping, we conducted the following experiment.

First, we computed the correlation between the slices of the even and odd subvolumes into which the input volume is split (see main text) without any registration. We then computed the correlation after non-rigid registration as implemented in N2N-REO. This was calculated by averaging: (a) the correlations between the even subvolume and the warped odd subvolume, and (b) the correlations between the odd subvolume and the warped even subvolume. For an additional comparison, we also evaluated the performance using rigid registration. The evaluation was carried out across the series of datasets generated from experimental Dataset 1 at various slice thicknesses (5 nm to 40 nm; see Section 3.3 in the main text).

The correlations were calculated as a function of spatial frequency using the Fourier Ring Correlation (FRC). The FRC was computed as described in the previous section, i.e. by averaging the FRC curves obtained across all slices in the volume. Fig. **S8** (left) displays the FRC between the subvolumes without warping (red curves) and with non-rigid warping as implemented in N2N-REO (green curves). For comparison, Fig. **S8** (right) shows the FRC obtained using rigid registration (green curves).

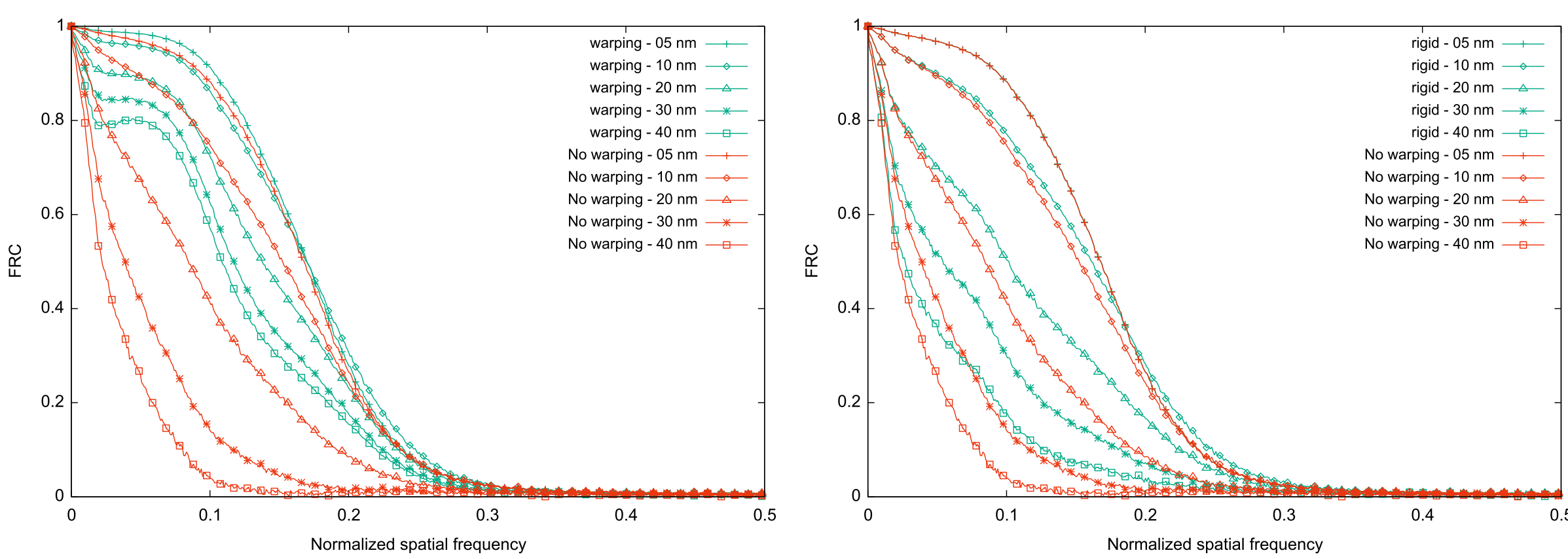

Figure S8: Fourier Ring Correlation curves obtained between even and odd subvolumes without warping (red curves), with warping based on the non-rigid registration involved in N2N-REO (green curves on the right plot) and with rigid registration (green curves on the left plot).

Fig. **S8** shows that non-rigid registration (green curves, left) improves, as expected, the FRC compared to unregistered subvolumes (red curves), specifically at low frequencies. However, at mid-to-high frequencies –where noise predominates–, the FRC remains close to zero. In the case of rigid registration, the low-frequency FRC improvement is much more subtle, while mid-to-high frequency values also remain near zero. Therefore, these results indicate that registration does not increase noise correlation.

Furthermore, the improved FRC curves with registration (green curves) are, at best, similar to the FRC obtained for the unregistered subvolumes at the smallest slice thickness (red curves for 5 nm), which represents the closest approximation to two independent views of the same scene in FIB/SEM. This confirms that registration does not introduce noise correlation beyond what is already present in the raw data.

Overall, these results demonstrate that noise at mid-to-high frequencies is uncorrelated and that registration –whether rigid or non-rigid– does not introduce spatial noise correlations, enabling Noise2Noise to operate correctly within N2N-REO.

S5. Denoising in other vEM modalities.

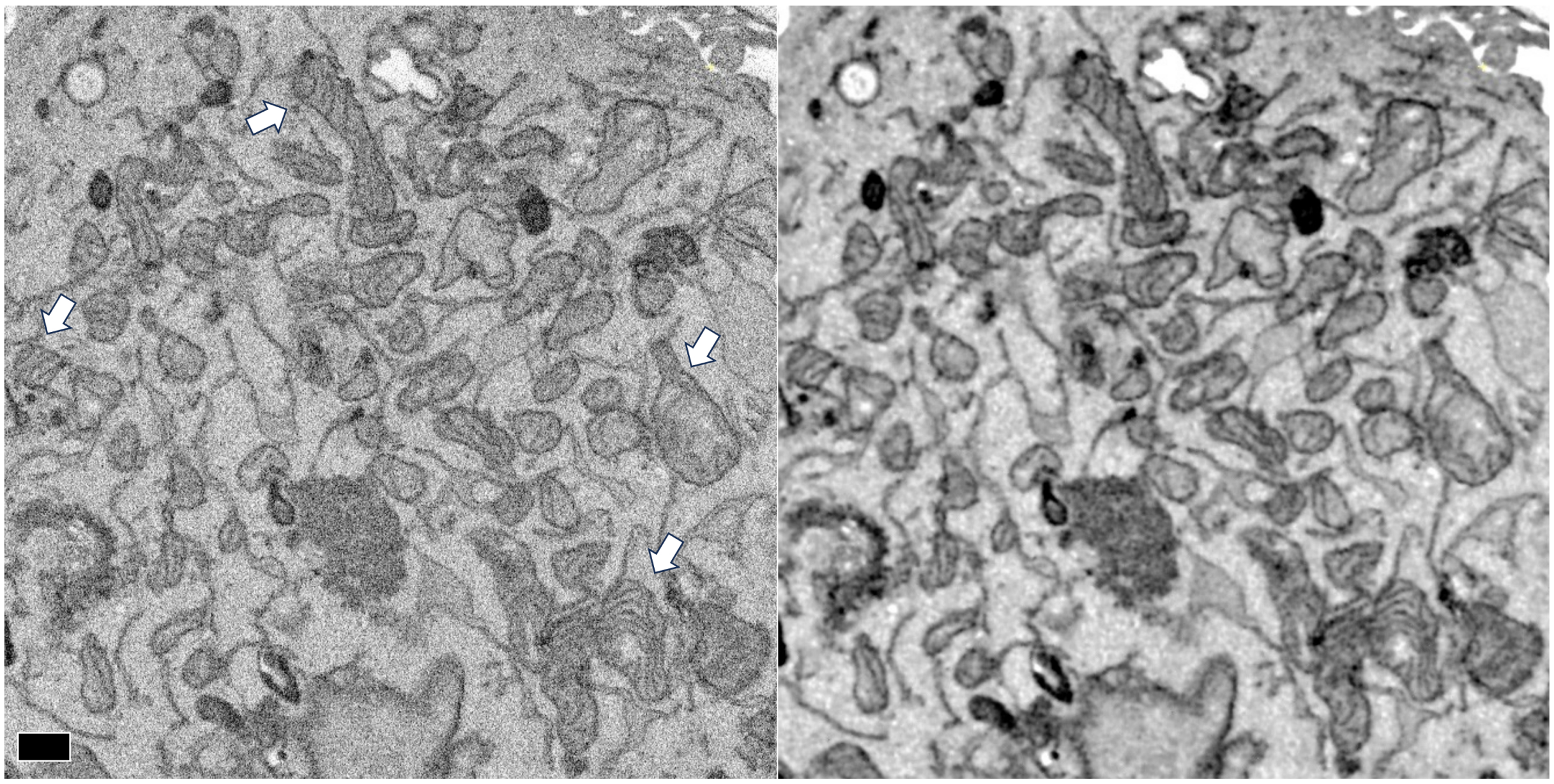

Figure S9: Motion-corrected N2N denoising (N2N-REO) applied to Serial-Block-Face Scanning Electron Microscopy (SBF/SEM) of chemically fixed HeLa cells, with slice thickness 50 nm. A slice of the original dataset (left) and after denoising (right) is shown. Dataset available at EMPIAR, ID 10478 (https://www.ebi.ac.uk/empiar/EMPIAR-10478/). White arrows mark areas of interest where the cleanliness and signal sharpness after denoising is remarkable. Bar: 500 nm.
